# L’oncologie médicale, nouvelle spécialité en Afrique?

**DOI:** 10.11604/pamj.2017.27.36.9531

**Published:** 2017-05-11

**Authors:** Sami Aziz Brahmi, Fatima Zahra Ziani, Youssef Seddik, Said Afqir

**Affiliations:** 1 Service d’Oncologie Médicale, Centre Hospitalier Mohammed VI, Oujda, Maroc; 2 Service d’Oncologie Médicale, Centre Hospitalier Hassan II, Fès, Maroc

**Keywords:** Oncologie médicale, Afrique, multidisciplinarité, Medical oncology, Africa, multidisciplinarity

## Abstract

Cancer is a major public health problem in Africa. Advances in the treatment of cancers over the last decade are undeniable. Multidisciplinary approach is essential for improved patient's management. Medical oncology is a recently-recognized speciality in Africa Indeed, many African countries do not have doctors or a sufficient number of doctors qualified to practice in this medical specialty. The fight against cancer in Africa involves oncology speciality training and the development of curricula in order to ensure optimum patient management.

## Aux editeurs du Journal Panafricain de Médecine

La base pour la création de l'oncologie médicale comme spécialisation a été posée en 1965 au moment de la fondation de l'ASCO (American Society of Clinical Oncology). Il s'agit de la spécialisation dont le domaine d'expertise concerne les traitements médicaux des cancers à savoir la chimiothérapie, les thérapeutiques ciblées et l'hormonothérapie. Le Maroc a formé 80 oncologues médicaux depuis l´introduction de cette discipline en 2004. Au cours des dernières décennies, on a assisté à des progrès rapides de la technologie médicale et des connaissances fondamentales de la biologie des cellules cancéreuses avec des retombées dans divers secteurs de l'oncologie à la génétique, le dépistage, le diagnostic précoce, les classifications et le traitement global du cancer. Ce développement a, en outre, permis une approche multidisciplinaire plus coordonnée du traitement spécifique de la tumeur et a fait naitre la volonté de formaliser la formation des oncologues à partir d'une série de directives ou selon un curriculum dans plusieurs spécialités majeures comme la chirurgie, la radiothérapie et l'oncologie médicale. En Afrique, le cancer est devenu un problème de santé public majeur. En 2012 on a estimé le nombre de nouveaux cas de cancer à 847 000 (soit un taux de 6.0% à l'échelle mondiale) et 591 000 de décès par cancer (soit un taux de 7.2% à l'échelle mondiale) [1]. Le nombre de nouveaux cas augmentera de 70% entre 2012 et 2020, une croissance plus rapide que n'importe quelle région au Monde [2]. Plus de 95% des patients atteints de cancer dans les pays africains sont diagnostiqués à un stade avancé, le retard de diagnostic pour ces patients est du au manque de la sensibilisation de la population et à l'inexistence de centre spécialisé avec du personnel bien formé [3]. Pour combattre ce fléau une approche multidisciplinaire est essentiel dans laquelle l'oncologue médical occupe une place de choix. Peu de données sont disponibles sur la spécialisation d'oncologie en Afrique. Dans une étude réalisée par l'European Society of Medical Oncology, qui a fait l'état des lieux de cette spécialité dans les pays en voie de développement incluant 4 pays Africains (Maroc, Afrique du Sud, Lybie et Egypte) on a dénombré moins de 10 oncologues par million d'habitant dans ces pays [4]. La situation en Afrique subsaharienne semble plus alarmante. En plus, selon la même étude, la durée de formation semble plus longue en Afrique par rapport à l'Europe. En outre, il existe une hétérogénéité importante en termes de cursus, d'années de formation de modalités de certification entre ces différents pays. A l'inverse l'oncologie médicale est reconnue comme une spécialité à part entière dans la plupart des pays développées. Il existe par ailleurs une volonté à uniformisé le cursus de formation en oncologie médicale aux Etats Unis et en Europe. Ceci à aboutit à l'élaboration de Recommandations pour un curriculum global unique en Oncologie Médicale par deux sociétés savantes ; la Société Européenne d'Oncologie Médicale ESMO (European Society for Medical Oncology) et la Société Américaine d'Oncologie Clinique ASCO (American Society of Clinical Oncology) [5]. L'objectif principal de ces recommandations est d'améliorer la qualité du traitement et des soins au patient, de fixer un standard de compétence clinique pour la pratique de l'oncologie médicale et d'encourager la formation continue pour l'excellence professionnelle au cours d'une vie de pratique médicale. Le futur de la prise en charge des patients atteints de cancer en Afrique passe obligatoirement de la formation de médecins compétents en oncologie médicale, le rôle des sociétés savantes s'avèrent essentiel pour l'élaboration de directives et recommandations dans ce sens.

## Conclusion

La plupart des pays Africains ne disposent pas de médecins qualifiés ou au nombre optimal en oncologie médicale. La formation de médecins qualifiés dans cette spécialité représente un chantier d'une extrême importance dans la lutte contre le cancer en Afrique.

## Conflits d’intérêts

L' auteur ne déclare aucun conflit d'intérêt.
